# Broad proteomics analysis of seeding-induced aggregation of α-synuclein in M83 neurons reveals remodeling of proteostasis mechanisms that might contribute to Parkinson’s disease pathogenesis

**DOI:** 10.1186/s13041-024-01099-1

**Published:** 2024-05-22

**Authors:** Casey J. Lumpkin, Hiral Patel, Gregory K. Potts, Shilpi Chaurasia, Lauren Gibilisco, Gyan P. Srivastava, Janice Y. Lee, Nathan J. Brown, Patricia Amarante, Jon D. Williams, Eric Karran, Matthew Townsend, Dori Woods, Brinda Ravikumar

**Affiliations:** 1 AbbVie, Cambridge Research Center, 200 Sidney Street Cambridge, Cambridge, MA 02139 USA; 2 Discovery Research, AbbVie Inc, 1 North Waukegan Rd, North Chicago, IL 60064 USA; 3 Excelra Knowledge Solutions Pvt Ltd, Uppal, Hyderabad India 500039; 4 Genomics Research Center, Computational Biology Neuroscience, AbbVie, Cambridge Research Center, 200 Sidney Street, Cambridge, MA 02139 USA; 5 Data & Statistical Sciences, AbbVie, Cambridge Research Center, 200 Sidney Street, Cambridge, MA 02139 USA; 6 Biotherapeutics, AbbVie Bioresearch Center, 100 Research Drive, Worcester, MA 01605 USA; 7 Laboratory of Aging and Infertility Research, Department of Biology, Northeastern University, Boston, Massachusetts USA

**Keywords:** Parkinson’s disease, M83 mouse model, Total and phospho proteomics

## Abstract

Aggregation of misfolded α-synuclein (α-syn) is a key characteristic feature of Parkinson’s disease (PD) and related synucleinopathies. The nature of these aggregates and their contribution to cellular dysfunction is still not clearly elucidated. We employed mass spectrometry-based total and phospho-proteomics to characterize the underlying molecular and biological changes due to α-syn aggregation using the M83 mouse primary neuronal model of PD. We identified gross changes in the proteome that coincided with the formation of large Lewy body-like α-syn aggregates in these neurons. We used protein-protein interaction (PPI)-based network analysis to identify key protein clusters modulating specific biological pathways that may be dysregulated and identified several mechanisms that regulate protein homeostasis (proteostasis). The observed changes in the proteome may include both homeostatic compensation and dysregulation due to α-syn aggregation and a greater understanding of both processes and their role in α-syn-related proteostasis may lead to improved therapeutic options for patients with PD and related disorders.

**Supplementary Information:**

The online version contains supplementary material available at 10.1186/s13041-024-01099-1.

## Introduction

The deposition of misfolded proteins is a characteristic pathological feature associated with dozens of human neurodegenerative diseases including Parkinson's disease (PD). PD belongs to a group of related neurodegenerative disorders called synucleinopathies, in which the primary pathology is the intracytoplasmic accumulation of α-synuclein (α-syn) typically in neurons and, in some cases, glial cells [1]. In PD, fibrillar α-syn aggregates appear in the form of Lewy bodies (LB) and Lewy neurites (LN) primarily in the substantia nigra pars compacta resulting in the loss of dopaminergic neurons [2]. While most cases of PD are considered idiopathic, a genetic factor has been implicated in 5-10% of patients diagnosed [3]. The first genetic cause of PD identified was a point mutation (A53T) in the SNCA gene that encodes α-syn [4]. Since then, both point mutations and gene multiplications (duplication and triplication) of the SNCA gene have been reported in patients presenting with early onset PD [5, 6]. The exact function of α-syn, a highly expressed, 14kDa intrinsically disordered protein, however, remains poorly understood [7]. Under pathological conditions, α-syn monomers misfold, aggregate and spread in a prion-like manner between interconnected neurons [8, 9]. The insoluble protein is thought to contribute to disease either by gain of an unknown novel toxic function or by loss of a normal endogenous function. Exogenously added misfolded α-syn can act as a template to initiate the misfolding and aggregation of endogenous α-syn in both cellular and animal models of PD [10–12]. Analysis of LBs suggest that phosphorylation of α-syn at serine 129 (pS129) is the dominant post-translational modification and is typically enriched in the detergent-insoluble fraction of PD cell and animal tissue lysates [13, 14]. Several studies have attempted to characterize other α-syn modifications and distinct protein components of LBs using mass spectrometry (MS)-based proteomics approaches to understand the process of LB formation and its contribution to disease [15–18].

In order to fully understand the underlying cell biology and pathogenesis of PD, many *in vitro* and *in vivo* models have been established capitalizing on some of the genetic causes linked to familial PD [19]. One such model is the M83 transgenic mouse line, which overexpresses the human form of A53T α-syn under the mouse PrP promoter [20]. The homozygous M83 mice start to develop dramatic motor symptoms at 8 months of age, and this coincides with the appearance of α-syn inclusions around 7 months of age. The hemizygous M83 mice also develop the same phenotype but have a much later age of onset [20]. The α-syn inclusions in the M83 mice are found in both neuronal cell bodies and processes and recapitulate many of the same biochemical and histological properties of human α-syn inclusions including positive staining with pathologically exclusive α-syn antibodies, the presence of detectable ubiquitin modifications, positive Gallyas silver staining, detergent insoluble high molecular mass α-syn aggregates, and α-syn fibrils that measure 10–16 nm wide [20]. Ultrastructural analysis of brain sections from M83 mice show significant axonal degeneration and the presence of α-syn inclusions in these neurons [20]. The development of α-syn inclusions in M83 mice can be rapidly expediated with the intracerebral introduction of pathological α-syn purified from PD tissue or recombinant α-syn pre-formed fibrils (PFFs) generated *in vitro* [11]. M83 mice injected before the onset of symptoms, at 2-5 months, show central nervous system (CNS)-wide α-syn pathology including LBs and LNs as soon as 30 days post injection (dpi) [11]. Spreading of pathological α-syn inclusions in CNS regions distant from the site of injection is also evident as well as shortened lifespan [11].

Cellular processes that lead to and are disrupted by α-syn fibrillization, aggregation and formation of LBs and LNs induced by PFF inoculation have been previously studied *in vitro* [10, 12]. Such *in vitro* models are essential to understand the biology behind disease pathogenesis and for genetic and small molecule-based drug discovery approaches looking for modulators of α-syn aggregation and pathology. It has been shown that the treatment of non-transgenic (wild-type) embryonic primary mouse neurons with PFFs generated from either full-length or truncated recombinant α-syn can recapitulate the formation of α-syn inclusions resembling those found in human synucleinopathies [12]. In these conditions, small insoluble α-syn aggregates develop starting at day 4 and continuing through day 7 post-PFF treatment followed by the formation of LN-like structures by day 10 and eventually LB-like aggregates by day 14 and beyond [12]. Accumulation of the pathological α-syn aggregates coincides with loss of synaptic proteins and a concomitant decrease in functional connectivity of the neurons.

In depth proteomic analysis represents a powerful approach for deciphering disease-dependent changes in model systems and tissues from human patients. Characterization of insoluble α-syn aggregates isolated from primary neurons post-PFF treatment have identified proteins that were also found in the insoluble fraction of human brain with synucleinopathy providing confidence on the use of such in vitro models for research [16]. More recently, proteomic analysis of extracted, late-stage, insoluble α-syn inclusions from day 14 and day 21 post-PFF treatment of isolated primary neurons from non-transgenic mice was employed to further characterize LB formation in culture. This led to a more extensive understanding of the underlying cellular processes and molecular dyshomeostasis that contribute to LB formation and decline in neuronal health [21].

In this study, we used quantitative total proteomics and phospho-proteomics to extensively characterize temporal changes in the total and detergent-insoluble protein fractions isolated from neurons from M83 transgenic mice treated with recombinant α-syn PFF. We further employed protein-protein interaction-based network analysis to define the biological mechanisms altered due to α-syn aggregation to get a comprehensive understanding of specific mechanisms that can be targeted for rational drug design. Our results show broad changes in several key biological processes that may contribute to or be disrupted by the formation of α-syn aggregates over time. We specifically identified enrichment of several mechanisms regulating cellular proteostasis including changes in several RNA binding proteins. It is our understanding that this is the first time that total proteomics and phospho-proteomics have been used to capture aggregation specific changes in a PD primary neuronal culture model. Having a better understanding of the cellular landscape of primary neurons directly from the M83 transgenic mice will help us to improve the therapeutic relevance of the model and contribute to a synchronized translation from *in vitro* to *in vivo* work.

## Results

### Dose- and time-dependent formation of α-syn aggregates by addition of recombinant α-syn PFFs in M83 mouse primary neurons

**Fig. 1:**
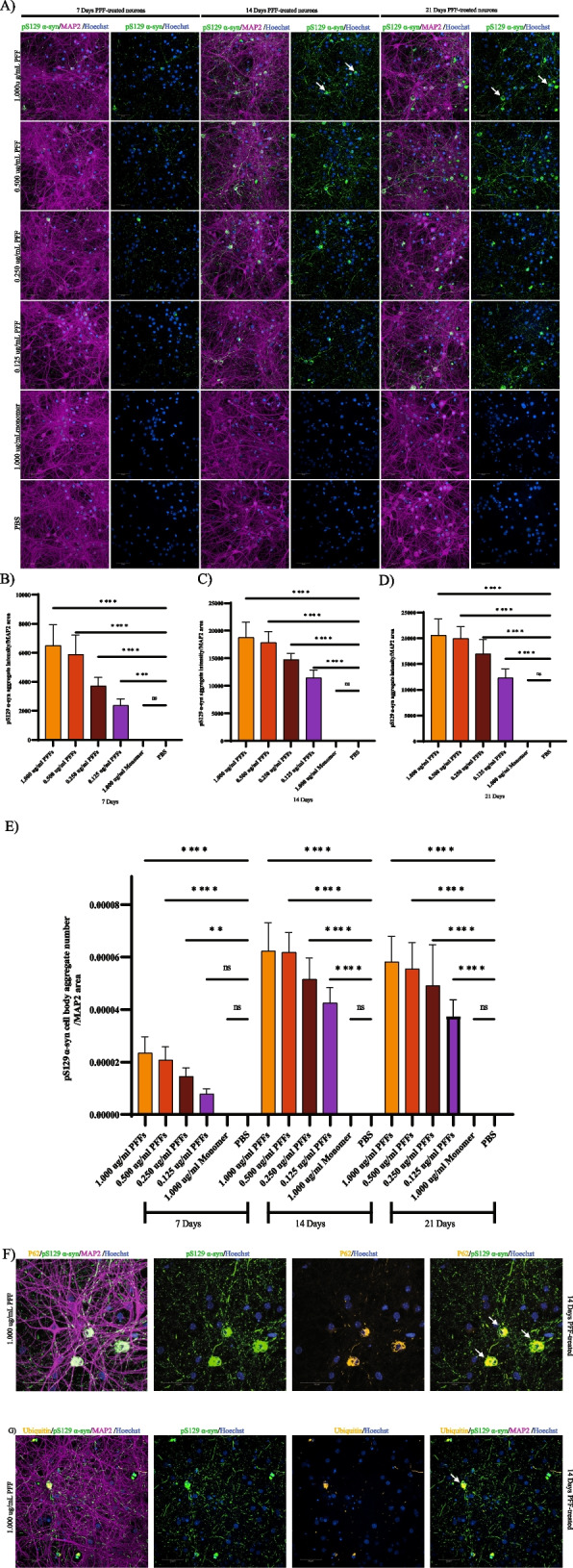
Characterization of α-syn aggregation in neurons isolated from M83 mice. **A** Recombinant human α-syn PFFs were added to DIV7 M83 neurons at 1.000,0.500, 0.250 and 0.125 ug/ml concentrations along with 1.000 ug/ml of α-syn monomer and PBS control. Cells were fixed at 7-, 14- and 21-days post-treatment. Small puncta of pS129-α-syn inclusions (green) were initially detected in neurites 7 days after α-syn PFF treatment. At 14 and 21 days of PFF treatment, pS129-α-syn aggregates become more elongated and some were also found in cell soma shown by arrows. **B**-**D** Quantification using high-content image analysis showed significantly higher total pS129-α-syn aggregate formation at 7 (**B**), 14 (**C**), and 21days (**D**) of PFF treatment in a dose-dependent manner and no aggregation was observed with PBS or α-syn monomer treatment. (*N*=6 replicates). Data are mean±SD ****p<0.0001 (Ordinary one-way ANOVA). **E** Immunofluorescence quantification suggested that the number of aggregates in the cell soma were higher at 14 and 21 days after PFF treatment compared to 7 days. **F**-**G** pS129-α-syn aggregates were positive for autophagy markers p62 and ubiquitin (orange). Neurons were stained with MAP2 (purple) and nucleus with Hoechst (blue). Scale bars, 50 μm

To understand the molecular and biological alterations associated with α-syn aggregation, we established a seeding-based model using primary cortical neurons from M83 transgenic mice that express the human A53T mutant (hA53T) α-syn under the mouse PrP promoter. The addition of exogenous α-syn PFFs can robustly induce aggregation of endogenous wild-type or overexpressed α-syn. Using a previously published and widely used protocol, we generated recombinant human α-syn fibrils [12]. We first evaluated the extent of aggregation of the endogenous hA53T α-syn in the M83 neurons after treatment with α-syn PFFs by staining for α-syn phosphorylated at serine 129 (pS129). Cortical neurons isolated from M83 transgenic mice were treated at days *in vitro* (DIV) 7 with human α-syn PFFs at 1, 0.5, 0.25 and 0.125 µg/ml concentrations, α-syn monomer at 1 µg/ml, and PBS vehicle control. After treatment for 7, 14, and 21 days, cells were fixed with methanol to remove soluble protein and immunostained for pS129 positive α-syn aggregates, microtubule-associated protein 2 (MAP2), a neuronal marker, and nuclei stain (Fig.S1 A.) Small puncta of pS129-α-syn aggregates were detected in neurites after 7 days of PFF treatment (Fig. 1 A). After 14 and 21 days of PFF treatment there was an overall increase in pS129-α-syn aggregate formation, and some aggregates were visible in the cell soma (arrow, top right, Fig. 1A). At all timepoints we observed a clear dose-dependent increase in pS129-α-syn aggregation with increasing concentrations of PFFs (Fig. 1B-D). The number of aggregates in the soma increased from 7 to 14 days after PFF treatment but did not increase further at 21 days after PFF treatment (Fig. 1E). We did not observe overt toxicity at any of the three timepoints as we did not observe significant changes in MAP2 area (Fig.S1 B). Furthermore, we observed a significant reduction in pS129-positive α-syn aggregates when we knocked down SNCA using pooled siRNAs against human SNCA (Fig.S1 C and D). The hyperphosphorylated α-syn aggregates in the soma appear condensed and resemble LBs in human PD brains and are also positive for p62 and ubiquitin consistent with previously published literature (Fig. 1F and G) [16, 22–24]. Likewise, we did not observe a large difference in α-syn aggregation between 0.5 µg/ml and 1 µg/ml of PFFs suggesting saturation around the 1 µg/ml PFF concentration. Thus, we observed a dose- and time-dependent increase in α-syn aggregation, with peak aggregation after 14 days of PFF treatment.

### Quantitative total and phospho-proteomic analysis in total lysates from M83 neurons treated with α-syn PFFs

**Fig. 2:**
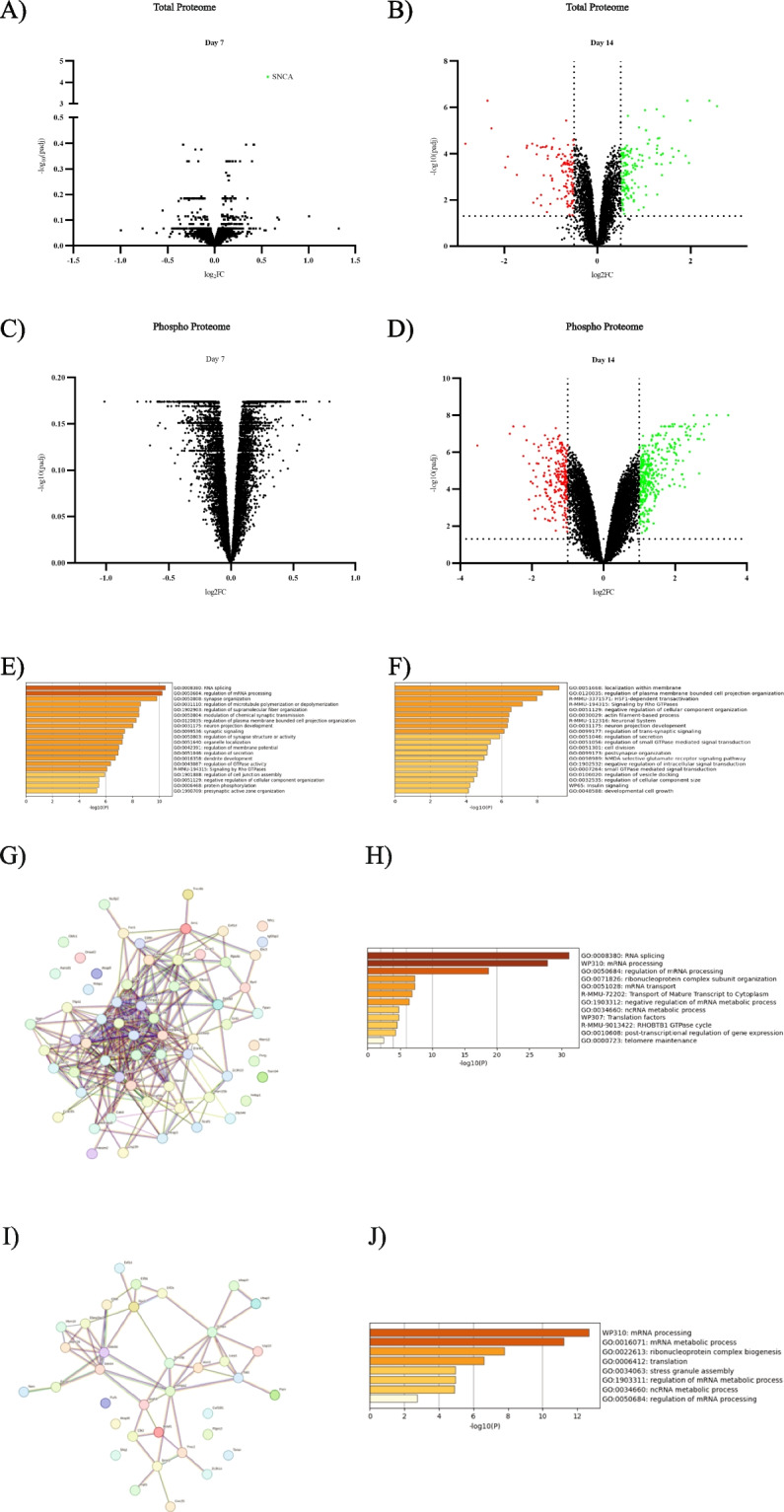
Temporal proteomic and phospho-proteomic analysis in the total lysates of M83 neurons with α-syn aggregation. **A**-**D** α-syn PFF mediated changes in protein expression in total lysates of M83 neurons were evaluated at 7-, and 14-days post-treatment using TMT-MS. Changes were plotted on a volcano plot with log fold changes on x-axis and negative log of *p* values on y-axis. Statistically significant changes were determined based on a false discovery rate of 0.05. Red dots indicate proteins significantly decreasing, green dots indicate proteins significantly increasing, and black dots indicate proteins that did not change. On day 7, no significant changes were noted in protein expression in proteomics (**A**) or phospho-proteomics (**C**). At day 14, a total of 135 proteins in the proteomics (**B**) and 633 phosphopeptides in the phospho-proteomics (**D**) showed statistically significant changes (**E**-**F**) Enrichment analysis using Metascape was performed based on the proteomics and phospho-proteomics data from the DIV14 post-PFF treatment timepoint. Processes related to RNA metabolism and microtubule organization were identified based on upregulated proteins (**E**) and HSF1 and actin-based processes were identified in the analysis of downregulated proteins (**F**). **G**-**J** Overlap evaluation with 1542 manually curated RNA binding proteins led to identification of 64 upregulated and 36 downregulated proteins. Subnetworks were created using String database based on these upregulated (**G**) or downregulated (**I**) proteins. Identification of mRNA splicing, transport, and metabolism was found in proteins that were upregulated (**H**). Identification of mRNA processing, mRNA metabolic process, and ribonucleoprotein complex biogenesis and translation was found in the proteins that were downregulated (**J**)

We wanted to better understand α-syn aggregation-mediated molecular changes to define specific disease-relevant signatures that could be used to identify novel drug targets or potential disease biomarkers. MS-based quantitative proteomics is a powerful technology that enables understanding of cellular and molecular mechanisms of disease. To this end, we used tandem mass tag MS (TMT-MS) to perform total and phospho-proteomics in M83 primary neurons treated with recombinant PFFs over time [25]. Individual 11-Plex TMT-MS was used for total lysate analyses from two different timepoints: 7 days post-PFF treatment when small α-syn aggregates were observed predominantly in the neurites and 14 days post-PFF treatment when we saw LB-like α-syn aggregation that did not increase with further incubation time [25, 26]. We chose 1 µg/ml PFF with which we saw maximal pS129-α-syn soma aggregates and compared to samples treated with PBS vehicle control (Fig. 1 E). A high degree of overlap was observed between days for both proteins and phosphopeptides with 5523 proteins and 14,227 phosphopeptides being consistently identified at both timepoints (Fig.S2 A and B). Since there may be broad proteome changes due to maturity of the neuronal cultures over time, and potential increase of glial cells during the later time points, comparisons were made within each individual timepoint for consistency. Principal component analysis (PCA) within each individual time point for both total and phospho-proteomics showed good clustering of replicates and separation by treatment (Fig.S3 A-D). The total amount of aggregation increased substantially between 7 and 14 days post PFF treatment. Some changes were observed in phosphorylated peptides 7 days post-PFF treatment. However we observed differential expression of several total and phosphopeptides at 14 days post-PFF treatment compared to PBS treated group when using the cutoffs of log_2_ fold change | (Log_2_ FC) >0.5| and adjusted *p*-value (padj)< 0.05 (Fig. 2 A-D).

Therefore, we performed the follow up computational analysis using data from the day 14 post-PFF treatment timepoint. First, we performed an analysis using Metascape, an online gene annotation and analysis resource [27]. Metascape analysis of all the total and phospho upregulated proteins showed affected processes related to RNA metabolism and microtubule organization (Fig. 2 E), while heat shock factor protein 1 (HSF1) and actin-based processes were identified in the analysis of all downregulated proteins (Fig. 2F). The identification of RNA-related processes led us to further investigate RNA binding proteins (RBPs) in the dataset. When we specifically looked for overlap with the 1542 manually curated RBPs [28] we identified 64 and 36 RBPs that were found in the up and down regulated proteins respectively (Fig. 2 G and I). These proteins seem to show strong connectivity as seen in the subnetworks generated using String database, a database of known and predicted protein-protein interactions (Fig. 2G and I) [29]. We saw changes in pathways related to mRNA splicing, transport, and metabolism in the RBPs that were upregulated, while the downregulated RBPs showed changes in mRNA processing, mRNA metabolic process, ribonucleoprotein complex biogenesis and translation (Fig. 2H and J). Taken together, this suggests a broad remodeling of the proteome due to aggregation that spans several pathways including heat shock response, cytoskeletal mechanisms and pathways regulated by RBPs.

### Network analysis to identify pathways impacted by α-syn PFF-mediated changes in proteomics and phospho-proteomics

**Fig. 3:**
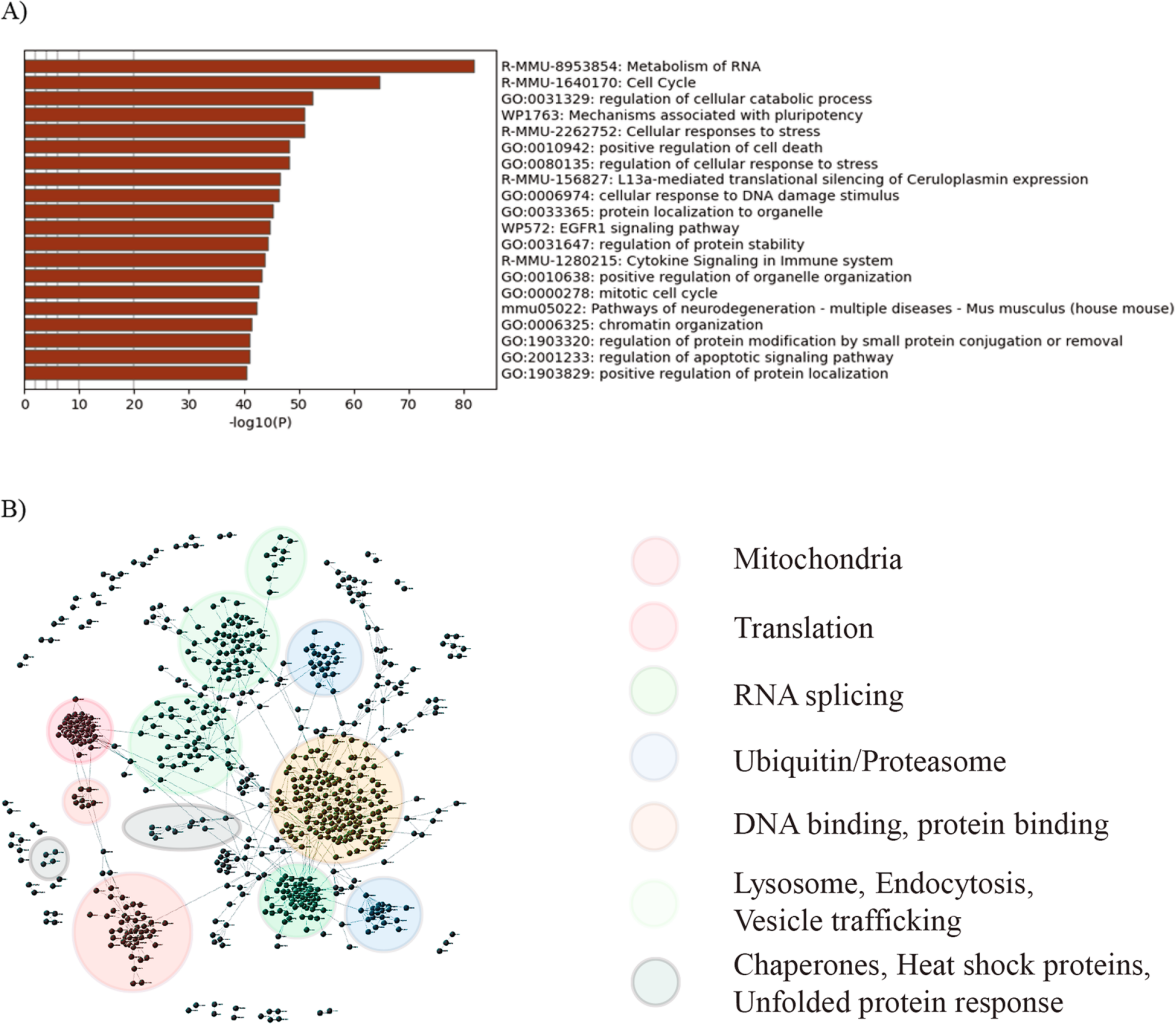
Network analysis for identification of pathways changing with α-syn PFF treatment in M83 neurons. **A** A total of 1,690 overlapping genes in the first order networks including proteins and phosphopeptides changing with PFF treatment at day 14 in proteomics (at the cut off of absolute value log FC>0.5) and phospho-proteomics (at the cut off of absolute value log FC>1) were identified. Metascape analysis of these genes showed enrichment of pathways regulating RNA metabolism and cell death, and cellular response to stress. **B** A zero-order network was generated from the 1,690 overlapping genes using internal network visualization tool. Pathways identified based on clustering of the 1,690 genes included various proteostasis mechanisms including splicing, ubiquitin related, proteasome, mitochondrial genes, translation, lysosome, endocytosis, unfolded protein response, vesicle trafficking and chaperone

We next performed a broader network analysis to figure out the significance of the total and phospho-proteome changes observed. Protein-protein interactions (PPI) are key to the proper functioning of proteins and the regulation of diverse cellular activities. PPI data can be used to generate *in silico* networks where each protein is represented by a node, and each interaction is represented with an edge between two nodes. Nodes with high degree of connectivity denote a hub. Direct interactions between seed genes represent the zero-order network, while interactions between seed genes and all nodes directly connected to the seeds represent the first-order network. These networks can be used as maps to identify specific disease-relevant clusters or changes that can elucidate the underlying molecular mechanisms misregulated during disease. We performed network analysis using the upregulated and downregulated total and phospho-proteomics at day 14 post-PFF treatment. Using an adjusted *p*-value < 0.05 and an absolute value |logFC > 0.5| and |logFC > 1.0| as thresholds for total and phospho-proteomics respectively, four input lists from the PFF vs PBS comparisons were used (Fig. 2A-D; Supplementary Table S1-S4). The proteins in the input lists were used as seeds to generate first-order PPI networks using ConsensusPathDB (CPDB), a resource which integrates public data from human, mouse and yeast into one meta-database with over 600,000 human protein interactions reported [30]. To decipher the biological mechanisms affected by PFF-mediated α-syn aggregation, we looked for common proteins that overlapped in the four first-order networks. There were 1690 proteins that overlapped and metascape analysis of these showed a very strong presence of pathways regulating RNA metabolism, cell death, and cellular response to stress (Fig. 3A). We next used an internally developed network-based visual analytics prototype to build a zero-order network using the 1690 overlapping proteins at a stringent cutoff of 0.999. Analysis of the network modules showed several clusters that correspond to different proteostasis mechanisms suggesting a broad dysregulation of proteostasis due to α-syn aggregation (Fig. 3B). The pathways identified included chaperones and heat shock proteins, ubiquitin-proteosome pathway, mitophagy, autophagy-lysosomal pathway, translation, unfolded protein response, vesicle trafficking and RNA splicing (Fig. 3B). Many of these pathways have evidence of being linked to PD pathology [31]. This is consistent with the notion that the regulation of proteostasis is essential in preventing protein aggregation and this tightly regulated balance of protein synthesis and clearance is disrupted during aging and neurodegeneration [32]. An in-depth analysis of proteins in the different clusters may yield novel drug targets and/or biomarkers of disease progression. Our data suggests a strong contribution of an imbalance in proteostasis towards α-syn induced pathology in M83 neurons.

### Proteomics analysis of the insoluble fraction of α-syn PFF treated M83 neurons

**Fig. 4:**
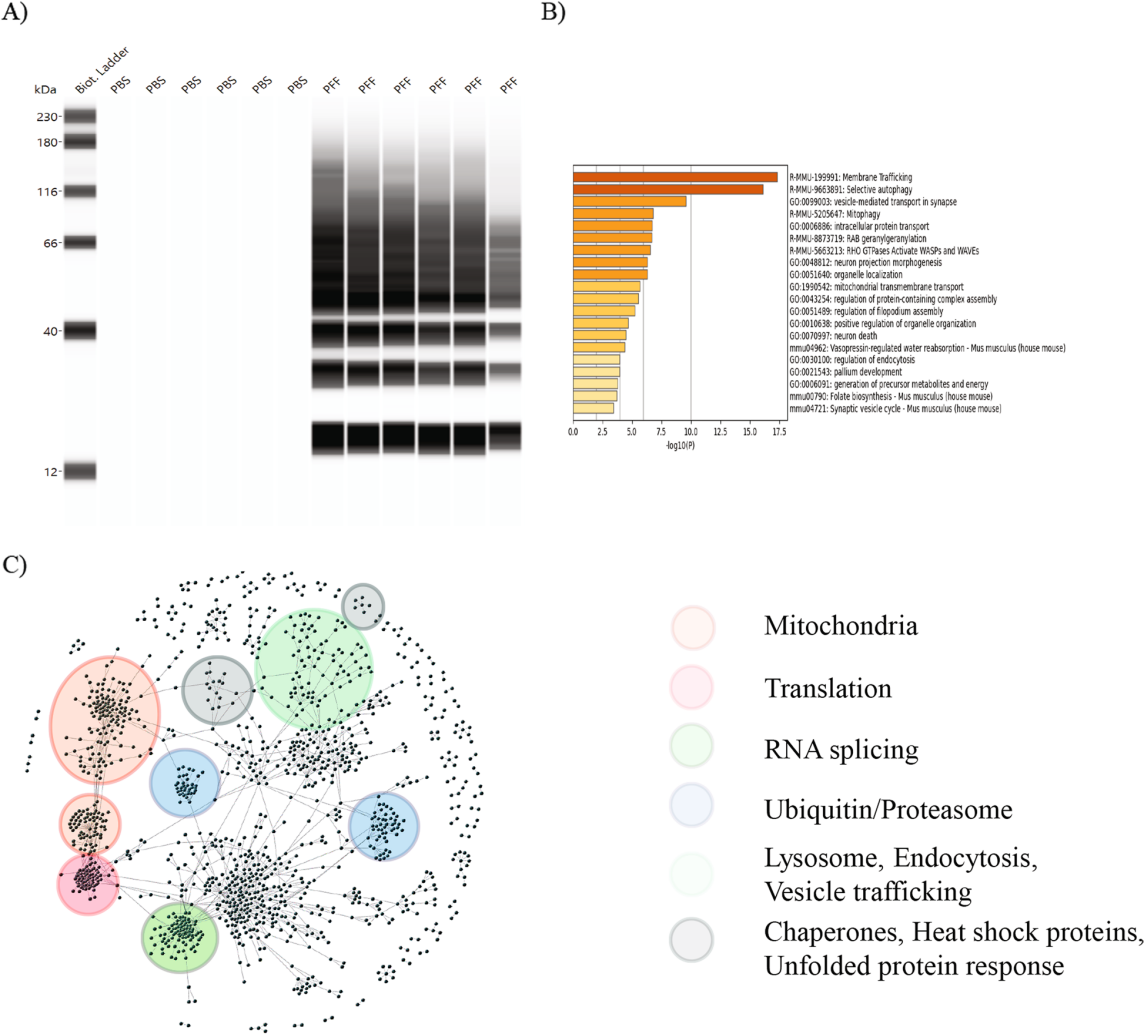
Proteomic analysis in the insoluble fraction of α-syn PFF treated M83 neurons. **A** M83 neurons treated with α-syn PFFs or PBS for 14± 1 and 21± 1 days were sequentially extracted with 1% Triton X-100 followed by 2% SDS. Western blot analysis showed significant enrichment of pS129 α-syn in the Triton X-100 insoluble fraction from PFF treated neuron samples. With PBS treatment, α-syn was extracted in 1% Triton X-100 fraction. **B** Insoluble fractions analyzed by Mass spectrometry identified 92 proteins that were changed in the total proteome with PFF treatment at logFC cutoff of ≥ 0.25. An enrichment analysis using metascape for these 92 proteins was performed, which identified membrane/vesicle trafficking, autophagy, endocytosis, and exocytosis mechanisms. **C** Based on a stringent cutoff of 0.999 in CPDB, a first order network of these 92 proteins including 1640 proteins and 4,738 interactors was generated. Based on this, a zero-order network was established using the internally developed network-based visual analytics tool to identify enriched pathways which are shown in the figure. The most enriched pathways in this network included the mitochondrial pathway, translation, ubiquitin related, lysosome, unfolded protein response, vesicle trafficking and mRNA splicing

MS-based approaches have been extensively used to characterize the detergent-insoluble proteome in cell lysates and tissues from animal models and human patients with different neurodegenerative diseases [12, 21, 33–35]. α-syn aggregates in wild-type primary mouse neuronal cultures treated with PFFs have been shown previously to be resistant to mild detergent treatment, like LBs isolated from human PD patient brain samples [16, 21, 36]. To profile disease-specific protein changes in the detergent-insoluble extracts, we performed a label-free quantitative proteomic analysis using M83 primary neuron lysates from DIV14 and DIV21 post-PFF treatment when LB-like α-syn aggregates are most pronounced. Based on the protocol described previously, we performed sequential extraction with 1% Triton X-100 and 2% SDS in M83 neuron lysates treated with PFFs or PBS for 14± 1 and 21± 1 days to isolate the insoluble fraction [12]. We confirmed by western blot analysis that pS129-α-syn is enriched upon PFF treatment (Fig. 4A) in the Triton X-100 insoluble and SDS extractable fraction. In the PBS treated neurons, α-syn was completely extracted in the Triton X-100 fraction. We then proceeded to analyze the samples by MS to identify proteins enriched with insoluble α-syn aggregates. Using a logFC of ≥ 0.25 and padj<0.05, 92 proteins were found to be enriched in the PFF treated insoluble fraction by total proteomic analysis (Supplementary Table S5). Similar to the total and phospho-proteome analysis performed on the total lysates, we first performed an analysis using metascape for the 92 proteins. Interestingly the top identified mechanisms were involved in membrane/vesicle trafficking and selective autophagy. Identified gene sets included mitophagy, mitochondrial transport, RHO GTPases activate WASP and WAVE and RAB geranylgeranylation (Fig. 4B). To further identify the broad molecular signature behind these changes, we built a first order interactome using a stringent cutoff of 0.999 in CPDB. Visualization of this network containing 1328 proteins and 3621 interactions using internally developed network-based visual analytics tool identified modules involved in proteostasis. Pathways in this network included the mitochondrial pathway, proteins involved in ubiquitin-proteasome pathway, vesicular trafficking, and mRNA splicing (Fig. 4C). The data from the insoluble proteome suggest changes that may also affect the solubility and re-localization of broader cellular proteins besides α-syn.

### Modulation of Larp1 affects α-syn aggregation in M83 primary neurons

**Fig. 5:**
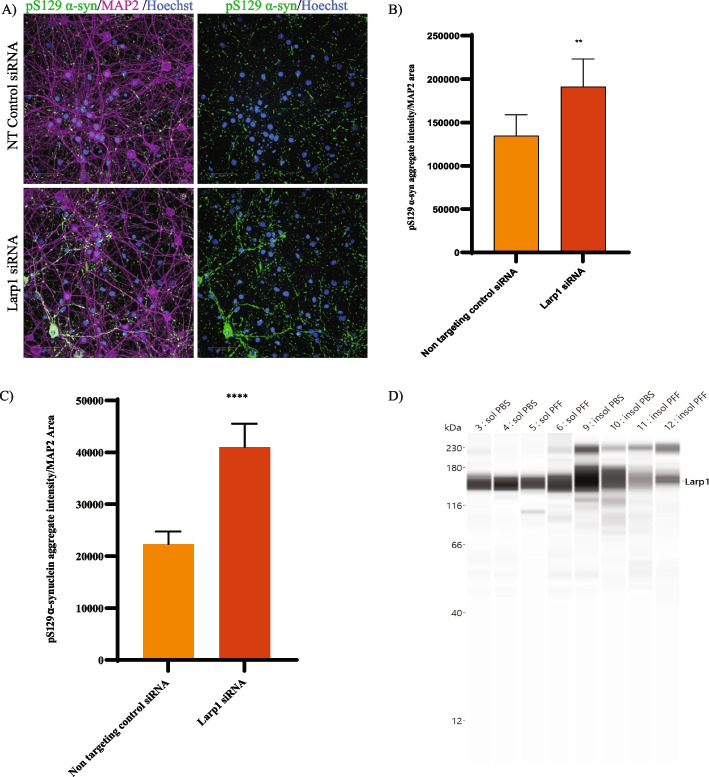
Modulation of Larp1 affects α-syn aggregation in M83 primary neurons. **A**-**B** The effect of Larp1 knockdown was evaluated on α-syn aggregation in the M83 seeding model. **A** Shows representative images with non-targeting control siRNA and LARP1 siRNA treatment in M83 neurons stained with pS129 α-syn (green), MAP2 (purple) and nucleus with Hoechst (blue). Scale bars, 50 μm. and (**B**) shows quantification using high-content image analysis, which indicated that knockdown of Larp1 significantly increased PFF-induced pS129 α-syn aggregation. (*N*=6 replicates). Data are mean±SD. **p=0.0066 (t-test). **C** Quantification of PFF-induced endogenous mouse α-syn aggregation in CD1 neurons also suggested that that knockdown of Larp1 resulted in significant increase in aggregation compared to non-targeting control siRNA. (*N*=6 replicates). Data are mean±SD. ****p<0.0001 (t-test). **D** WES analysis with Triton X soluble and insoluble fractions isolated from M83 mice with either PBS or PFF treatment showed enrichment of Larp1 in the PBS-treated samples but less enrichment in the PFF treated samples

We were intrigued to see several RBPs that were significantly changed in our phospho proteomics dataset. Specifically, the RBPs with phosphopeptides that were downregulated upon PFF-treatment seem to regulate ribonucleoprotein complex biogenesis and translation and some of these were also associated with stress granule assembly (G3BP1, ATXN2, TIAL1, LARP1, UBAP2L) [37, 38]. Identification of Larp1 in this cluster prompted further investigation since Larp1 is thought to be a key regulator downstream of mTOR involved in the stability and translation of 5’-terminal oligopyrimidine (TOP) motif comprising mRNAs, which encode proteins involved in the translation apparatus such as ribosomal proteins and translation factors [39]. TOP is one of the well-characterized cis-regulatory motifs for translational control located immediately downstream of the transcriptional start sites of mRNAs. We first tested the effect of Larp1 knockdown on α-syn aggregation in the M83 seeding model. Knockdown of Larp1 significantly increased PFF-induced pS129 α-syn aggregation (Fig. 5A and B). We also tested the effect of Larp1 knockdown in another α-syn aggregation model and saw similar effect. In this model we measured aggregation of endogenous wild-type α-syn after seeding with recombinant PFF (Fig. 5C). Interestingly, when we probed for Larp1 in the detergent-insoluble fraction, we found enrichment of Larp1 in the control (PBS-treated) conditions. This enrichment was reduced in the lysates from M83 neurons treated with PFF suggesting a change in solubility of Larp1 (Fig. 5D). A recent study reported that dephosphorylated SRSF2, a RBP involved in mRNA splicing, formed higher molecular weight detergent-insoluble oligomers [40]. Consistent with this report, we identified several phosphorylation sites on Larp1 that were significantly decreased after 14 days of PFF treatment (Table 1) and may explain the decreased Larp1 observed in the insoluble fraction. This suggests that phosphorylation of Larp1 may affect its solubility and oligomerization. The exact role of such modifications on Larp1 activity needs to be carefully examined and is beyond the remit of this paper. Previous studies have shown that TOP-containing genes not only code for the classical ribosomal and translation-related proteins but also other diverse proteins, some with lysosome-related and metabolism-related functions suggesting a role for Larp1 beyond translation regulation. We found 19/92 insoluble proteins that overlapped with predicted TOP-containing genes suggesting broad dysfunction of these proteins in PD [41].

**Table 1:**
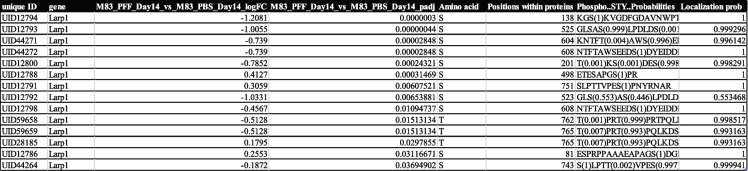
Phosphorylation sites on Larp1 that were significantly decreased after 14 days of PFF treatment in the total lysate samples from M83 neurons

## Discussion

Integrated omics approaches have been extensively used to characterize *in vitro* and *in vivo* PD models and PD patient samples. It is our understanding that this is the first time that total proteomics and phospho-proteomics have been used to capture aggregation specific changes in the M83 primary neuronal culture model. Although the progressive accumulation of aggregated α-syn in patients correlates with the appearance and progression of disease symptoms, the process linking α-syn aggregation to disease pathogenesis and neurodegeneration is still unclear. We employed quantitative proteomics and network analysis to gain insight into the molecular mechanisms that contribute to α-syn aggregation-induced changes in cortical neurons from M83 transgenic mice expressing the A53T human α-syn. Synthetic α-syn fibrils can induce a PD-like aggregation phenotype and accelerate pathology *in vivo* in M83 mice [11]. We found that α-syn aggregates initiated in the neurites as filamentous aggregates at day 7, and by day 14 post-seeding several condensed p62 and ubiquitin-positive aggregates were seen in the soma resembling LBs. Extending the incubation time to 21 days did not change the morphology or the proportion of these cell body aggregates, and this was in contrast to what was reported by Mahul-Mellier *et al.* using a wild-type seeding model [21]. This could be due to 3-5-fold overexpression of the human A53T transgene in neurons from M83 mice [20]. Consistent with the maximum pathology, we observed the most significant changes in the total and phospho-proteomics 14 days after PFF treatment.

In our dataset, we identified a large number of RBPs that change upon α-syn aggregation. RBPs play a key role in post-transcriptional gene regulation including splicing, RNA transport, translation, stability, metabolism, and RNA decay [42]. Our data detected a strong presence of RBPs that played a role in all these processes suggesting a widespread dysregulation of RBPs upon α-syn aggregation. Previous studies looking for genetic modifiers of α-syn toxicity as well as direct α-syn protein interactors found that after vesicle trafficking, proteins involved in mRNA metabolism more specifically RBPs and ribosomal subunits were the most identified [43–45]. It was also recently reported that α-syn directly associates with multiple mRNA-decapping proteins thereby modulating processing bodies (P-bodies), which are membraneless organelles that function in mRNA turnover and storage. P-body homeostasis and mRNA stability were altered in PD patient-derived iPSC-neurons and in PD-postmortem samples suggesting a direct role for α-syn in mRNA homeostasis [45]. More than 50% of RBPs in the eukaryotic proteome are expressed in the brain [46]. An interdependent network of RBPs has been hypothesized to regulate complex pathways in the CNS and alterations in this regulatory RBP-network could explain the dysfunction seen in different neurodegenerative and neuropsychiatric diseases [47] Not surprisingly, dysregulation of RBPs has been reported in diverse neurodegenerative diseases including frontotemporal dementia, amyotrophic lateral sclerosis, Huntington’s Disease (HD) and Alzheimer’s Disease (AD). RBPs were enriched in modules that correlated with AD pathology while aberrant interaction of mutant huntingtin oligomers with RBPs has also been reported [48–50]. In AD, RBPs have been found in the detergent-insoluble fraction and to co-aggregate with the microtubule associated protein tau, the pathological component of neurofibrillary tangles [33, 51]. Tau interacts with RBPs and promotes stress granule (SG) formation concomitantly accelerating tau aggregation [52, 53]. We identified components of SGs in our dataset including Larp1. The formation of SGs represents cells’ ability to cope with stress and regulate cellular translation. We believe that α-syn aggregation induces a broad change to the cellular proteome evident by enrichment of multiple proteostasis mechanisms. This suggests the contribution of a common pathway in related neurodegenerative diseases and offers the potential for targeting multiple diseases using a common therapeutic strategy. It will be important however, to see if specific RBPs regulating select RNA mechanisms are modulated in the different diseases. A recent study using RNA pulldown and MS identified disease subtype specific variations in the RNA-binding proteome of sporadic and progressive AD [54].

Our data adds to the extensive and carefully executed data sets looking at changes in the insoluble proteome over time in PFF induced endogenous mouse α-syn aggregation and Lewy body-like inclusion formation in wild-type primary mouse neuronal cultures [16, 21]. It is interesting to note that the insoluble proteomic changes we observed seem to be consistent with changes previously reported for PD – e.g., mitophagy, autophagy, vesicle mediated transport etc., [55]. These proteins might be enriched in the insoluble fraction by being sequestered into aggregates away from their normal location or due to a general upregulation and aberrant interaction with aggregates [24]. We found several overlaps of the insoluble proteins with the dataset reported by Mahul-Mellier *et al.*, suggesting common aggregation-mediated molecular changes [21]. PPI networks can be used to generate disease-specific maps and identify signatures that contribute to a particular phenotype. We built a broad PPI interactome using the pathology-specific total and phospho-proteomic changes and identified several clusters involving mechanisms regulating cellular proteostasis. More recently, Haenig *et. al.* used PPI-based interactome mapping to provide a network of neurodegenerative disease proteins [56]. This effort identified both distinct and interconnected disease modules suggesting both disease-specific and common mechanisms contributing to disease pathologies. Deeper understanding of these changes will provide novel therapeutic opportunities for targeting PD and related neurodegenerative diseases.

## Methods

### Preparation of primary neuron cultures

Primary cortical neurons were prepared from M83 heterozygous mice (B6;C3-Tg(Prnp-SNCA*A53T)83Vle/J; JAX number 004479). All animal experimental procedures were reviewed and approved by the Abbvie Institutional Animal Care and Use Committee (IACUC). Day 15.5+/-0.5 days embryos were removed from pregnant female and placed into 10cm dish with Ca and Mg free HBSS. Individual embryos were placed in a well of a 6 well plate containing 2-3 mls of Hibernation media (2% B-27 Supplement; 48% Hibernate-E media; 20% L15 media 30%CO2 independent media) on ice. Using a dissection microscope, the hippocampus and whole cortex were isolated and placed in a 15 ml conical tube with 10 mls of HBSS. HBSS was then aspirated, and neuronal isolation enzyme diluted in Ca/Mg free HBSS was incubated in a 30°C for 30 mins with gentle inversions every 5 mins. Cells were triturated and washed with DMEM/10% FBS and gently passed through a 70uM cell strainer. Cells were then counted and plated using plating media (DMEM/10% FBS/1X Penstrep). 3-6hrs after plating, media was completely removed and replaced with culture media (2% B27 plus 1% Glutamax in Neurobasal Plus media).

### Preparation of α-syn pre-formed fibrils (PFF)

Recombinant human α-syn PFFs were generated from monomeric starting material following an established protocol [57]. Briefly, recombinant human α-syn (1-140) at 6 mg/ml in 10 mM Tris, 50 mM NaCl, pH 7.6 was thawed on ice and then centrifuged at 15,000 x g for 10 min at 4°C to pellet out any preexisting higher molecular weight species. The supernatant was then collected, α-syn concentration measured, and conditions adjusted to 5 mg/ml α-syn in 10 mM Tris, 150 mM NaCl, pH 7.6. 0.5 ml α-syn was then aliquoted into individual, sealed, 1.7 ml Eppendorf tubes and placed in an Eppendorf thermomixer at 37°C, 1000 RPM for seven days. Successful fibrillization was confirmed by thioflavin t binding assay, sedimentation assay and imaging by negative staining transmission electron microscopy. α-syn fibrils were then aliquoted and stored at -80°C until use.

### Primary neuron culture treatment with human α-syn pre-formed fibrils (PFF) or PBS

Primary neurons were treated with the indicated concentration of PFFs (.125 ug-1 ug/ml for dose response and 1 ug for all subsequent proteomic samples) one time on DIV 7. 5uL aliquots of 5 mg/ml samples were thawed at room temperature and 245uL of PBS was added and mixed by gentle pipetting to bring to a concentration of 100 ug/ml. PFFs and PBS were then sonicated using a water bath sonicator and diluted to 2x in culture media (2% B27 plus 1% Glutamax in Neurobasal Plus media). 50% of culture media was removed from each well and replenished with either the 2x PFF or PBS culture media and returned to the incubator. Media was replenished every 3-4 days until cells were collected. For the time course (see Fig.S1 A.) Cells treated for 7 days with PFF were collected at DIV 14, cells treated for 14 days were collected at DIV 21 and cells treated for 21days were collected at DIV 28. All cells received a one time addition of PFF(7 day, 14 day and 21 day treatment nomenclature corresponds to how many days after the one time PFF treatment cells were collected)

Primary mouse M83 neurons were treated with 1uM accell Larp1 siRNA Smartpool or accell non-targeting siRNA pool, from dharmacon at DIV 5. α-syn PFFs were added at DIV 8 for 2 weeks at 0.5 ug/ml. For endpoints, one plate was fixed with methanol and stained for pS129 α-syn aggregates, MAP2 and nuclei number and the second plate was used for qPCR analysis to measure gene knockdown efficiency.

### Immunofluorescence protocol

After treatment, neuronal media was gently removed and cells were incubated with ice cold methanol for 20 mins at -20°C. Cells were washed 3x with dPBS (Thermofisher Scientific cat#14190144) and blocking buffer (4% Albumin Bovine Fraction V (BSA) Research Products International, 0.2% Triton in dPBS) was added to each well for 1 hr at room temperature. After sufficient blocking, blocking buffer was removed and primary antibodies (α-syn pS129 Cell Signaling Technology 23706 (1:3000); MAP2 Abcam ab5392 (1:2500) were diluted in blocking buffer and incubated overnight on an orbital shaker at 4°C. After primary antibody incubation, cells were washed 3x with dPBS. Alexa-flourophore-conjugated secondary antibodies were added to blocking buffer and filtered through Spin-X column centrifuged at 5000 RPM for 5 mins. Filtered secondary antibodies diluted in blocking buffer were added to each well. Cells were incubated at room temperature in the dark in secondary antibody on an orbital shaker for two hours. Cells were washed with dPBS 3x and nuclear stain was diluted in dPBS and added for 15 mins at room temperature. Cells were then washed with dPBS 3x and stored in dPBS at 4°C until imaging.

### Isolation of insoluble fraction

This protocol was originally published by Volpicelli-Daley [12]. Briefly, primary neurons plated at a density of 1,000,000 cells/ well in 6 well dishes were washed twice with dPBS. Cells were lysed in 250uL 1% Triton in TBS (1%Triton-X, 50mM Tris, 150mM NaCl pH 7.4) with 1X Halt Protease and phosphatase inhibitor. Using a cell scraper, cells were collected and placed in tubes on ice. Cells were sonicated on ice ten times at a pulse of 0.5 second on 0.1 second off with 20% amplitude. After sonication tubes remained on ice for 30 min. Cells were centrifuged at 100,000 x g for 30 mins at 4°C. The supernatant was collected from each sample and saved as the soluble fraction. A total of 250uL of 1% Triton in TBS was added to each pellet and subsequently sonicated ten times at a pulse of 0.5s on 0.1s off with 20% amplitude on ice. The sonicated pellets were then centrifuged down at 100,000 x g for 30 mins at 4°C. The supernatant was discarded and 80uL of 2% SDS buffer (2% SDS 50mM Tris, 150mM NaCl pH 7.4) was added and each pellet was resuspended. Samples were sonicated 15 times at 0.5s on 0.1s off pulse with 30% amplitude in a water bath sonicator. Samples were then immediately frozen at -80°C until further processing.

### Western blot

Protein concentrations were measured by micro-BCA assay (ThermoFisher Scientific 23235) and samples were normalized. Samples were run on the Protein Simple Wes instrument according to the manufacturer’s instructions. Depending on the size of the protein either the 2-40kDa, 12-230kDa or the 66-440kDa Separation Module was used along with either the Anti-Rabbit or Anti-Mouse Detection Modules. Primary antibodies used were pS129 α-syn (CST 23706) and Larp1 (CST 14763S). Briefly, Fluorescent Master Mix was added to diluted samples and samples were then heated at 95°C for 5 mins before loading into the cassette along with the antibodies, chemiluminescent substrate, and blocking reagent. The Wes instrument was run on default settings. Area under the curve (AUC) was quantitated from the resulting electropherograms. Samples were normalized to loading controls cofilin (CST 5175S), beta-actin (Licor 926-42210) or vinculin (abcam ab129002) and data graphed as protein/loading control.

### M83 total and phospho-proteomic sample prep

M83 mouse neurons were lysed in a buffer of 8M urea, 10 mM TCEP, 40 mM chloroacetamide, and 50 mM Tris supplemented with protease/phosphatase inhibitors. Protein concentrations for all treatments and timepoints were determined by protein BCA (Thermo Fisher Scientific, #23225), and equimolar protein concentrations were aliquoted, diluted to 1.5 M urea with 100 mM Tris, and digested with a trypsin/Lys-C combination enzyme mixture (Promega, V5071) in a 1:50 enzyme/substrate ratio overnight. Each sample was acidified to 0.1% TFA, desalted with 100 mg Strata-X polymeric reversed phase desalting columns (Phenomenex, #8B-S100-ECH), and dried down via a centrivap. Each timepoint’s 10 sample treatments were labeled with their own TMT 11-plex isobaric chemical tag set (Thermo Fisher Scientific, #A34808), reserving the 11^th^ channel for a “pooled” channel. This pooled channel consisted of a mix of small aliquots from the study’s 30 total samples, which served as a reference for normalization and cross-comparison of relative differences across the three TMT 11-plex sets. Once each set of TMT 11-plex peptides was mixed, quenched, and desalted a second time with Strata-X columns, each set was individually resuspended in 80% ACN 0.1% TFA and enriched for phosphopeptides using Qiagen Ni-NTA beads as described below. The flowthrough from Fe-IMAC enrichment contained non-phosphopeptides for proteome quantification. The three enriched phosphopeptide sets and three flowthrough proteome samples were each fractionated using a Waters Acquity UPLC. Peptides were separated using a linear gradient starting with aqueous 20 mM ammonium formate and increasing up to 20 mM ammonium formate in 80% ACN using a 2.1 x 100 mM UPLC column packed with 1.7 uM BEH C18 material (#186002352). Both phosphopeptide and proteome samples were collected into 15 total fractions, dried down via a centrivap, and resuspended in 0.1% formic acid.

### Phosphopeptide enrichment

Iron immobilized metal affinity chromatography (FeIMAC) was used to enrich phosphoserine, threonine, and tyrosine residues in the samples. First, samples were dried down and resuspended in 80% ACN and 0.15% TFA to prepare them for phospho-enrichment. Meanwhile, Ni-NTA magnetic agarose beads (Qiagen #36113) were cleaned with 40 mM EDTA and washed with water. Fe3+ was then chelated onto the NTA magnetic agarose beads for 30 mins at room temperature (RT) and 1350 RPM in a ThermoMixer. Beads were then washed in the same buffer that the samples were resuspended in (80% ACN and 0.15% TFA) and aliquoted evenly across all samples (500ul beads per mg of sample). FeIMAC beads were incubated with samples for 30 mins in a ThermoMixer at RT and 1350 RPM. Phosphopeptides were eluted thereafter with 50% ACN 0.7% NH_4_OH solution and immediately acidified with 4% FA. The eluate was dried down and resuspended in 0.1% formic acid and injected for MS analysis.

### M83 insoluble aggregate sample preparation

18 insoluble aggregate samples from PFF treated neurons and 18 samples from PBS treated neurons were processed for proteomics. Detergent clean-up and sample digestion was done with single-pot, solid-phase enhanced sample prep (SP3) and 96 well plate format robot assisted sample handling (Integra Assist Plus). First, 0.5M TCEP (Tris(2-carboxyethyl)phosphine), 0.5M CAA(Chloroacetamide) were added to each sample. Samples were reduced and alkylated in a covered shaker (Eppendorf ThermoMixer) at 1000 RPM for 30 mins at 37 °C. Meanwhile, equal amounts of Sera-Mag SpeedBeads (GE Healthcare, #45152105050250) and Sera-Mag carboxylate modified magnetic particles (GE Healthcare, #65152105050250) were combined, washed with water, and reconstituted with water in equal volume (SP3 beads). The SP3 beads (10ul), 0.5M DTT(Dithiothreitol), and 80% ethanol were added to each sample and mixed on the ThermoMixer at 1200RPM for 10 mins at room temperature to facilitate binding. The sample-bead mix was then washed three times with 80% ethanol. Trypsin/Lys-C combination enzyme mixture was added at a 1:50 enzyme/substrate ratio for an overnight digestion (1000 RPM, 37 °C, ThermoMixer). The next day, samples were acidified to pH 2 for C18 column-based sample clean up. Thereafter, samples were dried down and resuspended in 0.1% formic acid. 500 ng of sample was injected for MS analysis and the remaining used for phospho-enrichment. Insoluble aggregate samples were enriched for phosphopeptides as described above, and both insoluble proteome peptides and phosphopeptides were injected for MS analysis and quantified in label-free fashion.

### LCMS methods (total, phospho, and insoluble proteome)

Each TMT 11-plex’s 15 phosphopeptide and 15 proteome fractions were analyzed using a 120 min nano-LC MS data dependent acquisition (DDA) method. Peptides were separated using a Thermo Fisher Scientific Easy nanoLC 1200 with solvents 0.1% formic acid in water and 0.1% formic acid in 80% ACN. Peptides were separated linearly from 4% ACN to 45% ACN using an Easy Spray ES902 analytical column. Phosphopeptide or proteome fractions were analyzed using a Thermo Fisher Scientific Orbitrap Exploris 480 mass spectrometer, with survey MS scans collected at 120,000 resolving power and 350-1800 *m/z* scan range. A MS1 standard (100%) AGC target was selected with maximum injection times automatically calculated within a 2 second MS cycle time. Charge states 2-8 were considered for fragmentation with a 25 second exclusion duration. Peptides were selected with a 0.8 *m/z* quadrupole isolation window and fragmented with a 36% normalized collision energy. Orbitrap MS/MS resolving power was set to 45,000, with standard (100%) AGC targets. Phosphopeptide fractions were analyzed with a maximum injection time of 120 msec, while maximum injection times for proteome fractions were automatically calculated based on available cycle time. Insoluble aggregate proteomics and phospho-proteomics data was collected using an Evosep nano-LC running 44 min 30 SPD standard gradient methods and connected to a Thermo Fisher Scientific Lumos MS. The Lumos was run at 120,000 resolving power with a 350-1800 *m/z* scan range. A MS1 standard (100%) AGC target was selected with maximum injection times automatically calculated within a 3 second MS cycle time. Charge states 2-8 were considered for fragmentation with a 30 second exclusion duration. Peptides were selected with a 0.8 *m/z* quadrupole isolation window and fragmented with a 30% normalized collision energy. Orbitrap MS/MS resolving power was set to 7,500, with standard (100%) AGC targets. Phosphopeptide fractions were analyzed with a maximum injection time of 120 msec, while maximum injection times for proteome fractions were automatically calculated based on available cycle time.

### Proteomics data analysis

All TMT data and insoluble aggregate proteomics data were searched using MaxQuant, set with either TMT 11-plex chemical modifications or no modifications (insoluble aggregate phospho/proteomics). Enzyme digestion was specified as tryptic with a maximum of 2 missed cleavages. Chemical modifications considered for data searching included a fixed modification of carbamidomethylation of cysteines and variable modifications of methionine oxidation, acetylation of protein N-termini, and phosphorylation of serine, threonine, and tyrosine residues (for phospho-proteome MS data searches). All data was searched against a target-decoy Uniprot mouse proteome database containing both canonical sequences and protein isoform sequences, downloaded on September 20, 2020. Reporter ion MS2 spectra were used for TMT quantification with a precursor ion mass tolerance of 4.5 ppm and a fragment ion mass tolerance of 20 ppm. Whether for TMT or label-free quantification studies, protein groups and phosphosite quantitation MaxQuant tables were exported and filtered to consider quantifiable protein groups or phosphosites for >50% measurements were observed across all samples. Of those protein groups or phosphorylation sites that remained, missing values were imputed from the normal distribution of each samples’ protein or phosphosite quantitation. Protein group or phosphosite intensities from TMT studies were summed and median normalized to account for TMT mixing differences across the samples. Insoluble aggregate protein group LFQ and phosphosite intensities were also normalized by peptide amount loaded for LCMS.

### Proteomics differential abundance

From the quantile-normalized intensities for PFF- vs. PBS-treated samples within each timepoint/TMT run, additional downstream quality checks and analyses were performed on each of the TMT runs separately (each TMT run contained samples from a single timepoint: 7- or 14-days treatment). For each, we removed proteins only identified by site, potential contaminants, and reverse proteins. Of the remaining proteins, we kept proteins with at least one unique peptide and more than one peptide. We removed the pooled sample from each dataset and replaced all the zero normalized intensity values with ‘NA’ values. We excluded proteins if they contained missing values in > 20% of a timepoint’s conditions. For subsequent differential expression analyses, we used the Limma package in R. No covariates were used in the model. Within the differential expression results, we considered proteins with an adjusted *p*-value < 0.05 statistically significant.

### Phospho-proteomics analysis methods

From the quantile-normalized intensities for PFF- vs. PBS-treated samples within each timepoint/TMT run, additional downstream quality checks and analyses were performed on each of the TMT runs separately (each TMT run contained samples from a single timepoint: 7- or 14-days treatment). For each, we removed the pooled sample from each dataset. We removed phosphopeptides whose leading proteins were potential contaminants. We then replaced all the zero quantile-normalized intensity values with ‘NA’ values and excluded proteins if they contained missing values in > 20% of a timepoint’s conditions. Based on a PCAs excluding proteins with missing values, we detected and removed one outlier (M83_PFF_Day07_2) from downstream analyses. For subsequent differential expression analyses, we used the Limma package in R. No covariates were used in the model. No covariates were used in the model. Within the limma results, we considered phosphopeptides with an adjusted *p*-value < 0.05 statistically significant.

### Supplementary Information

Supplementary Material 1: S1. Experimental paradigm for α-syn PFF treatment in M83 primary neurons. (A) Schematic of α-syn PFF treatment time course in M83 primary neuronal cultures. α-syn PFFs, monomer and PBS were added to neurons at DIV7 for 7, 14 and 21 days. (B) No significant changes in neuronal viability observed by quantification of MAP2 area at 7, 14 or 21 days with α-syn PFF treatment compared to α-syn monomer or PBS treatment. (C) shows representative images with non-targeting control siRNA and human SNCA and mouse SNCA siRNA treatment in M83 neurons stained with pS129 α-syn (green), MAP2 (purple) and nucleus with Hoechst (blue). Scale bars, 50 μm. and (D) shows quantification using high-content image analysis, which indicated that knockdown of human SNCA and mouse SNCA reduced PFF-induced pS129 α-syn aggregation. (*N*=5 replicates). S2. Number of proteins identified at day 7 and day 14 in total and phospho-proteomic analysis in total lysates of M83 neurons treated with α-syn PFFs. The number of proteins identified at day 7 and day 14 are summarized with Venn diagrams in A (total proteins) and B (phosphor-peptides) A high overlap at two timepoints was observed for both total proteins and phosphor-peptides. A total of  5523 proteins (87.7%) and 14,227 unique phosphopeptides (57.5%) were identified at both day 7 and day 14 timepoints. S3. Principal Component Analysis (PCA) of each individual time point in total and phospho-proteomic analysis in total lysates of M83 neurons treated with α-syn PFFs. Principal Component Analysis (PCA) was conducted at day 7 and day 14 with PFF and PBS treatments for both total (A, B) and phospho-proteomics (C, D). We observed clustering of replicates and separation by treatment. S4. (A) WES analysis quantification of soluble and insoluble fractions isolated from M83 mice.  Data are mean±SD (B) M83 neurons treated with α-syn PFFs or PBS for 21± 1 days were sequentially extracted with 1% Triton X-100 followed by 2% SDS. Western blot analysis showed significant enrichment of pS129 α-syn in the Triton X-100 insoluble fraction from PFF treated neuron samples. With PBS treatment, α-syn was extracted in 1% Triton X-100 fraction.

Supplementary Material 2. 

## Data Availability

All data generated or analyzed during this study are included in this published article and its supplementary information files. Mass spectrometry proteomics data have been deposited to the ProteomeXchange Consortium PXID: PXD051424.

## Abbreviations

α-syn: α-synuclein
PD: Parkinson’s Disease
PPI: Protein-protein interaction
Proteostasis: Protein homeostasis
LB: Lewy bodies
LN: Lewy neurites
pS129: Phosphorylation of α-syn at serine 129
MS: Mass spectrometry
PFFs: Pre-formed fibrils
CNS: Central nervous system
dpi: Days post injection
hA53T: Human A53T mutant
MAP2: Microtubule-associated protein 2
DIV: Days in vitro
TMT-MS: Tandem mass tag mass spectrometry
PCA: Principal Component Analysis
HSF1: Heat shock factor protein 1
RBPs: RNA binding proteins
CPDB: ConsensusPathDB
TOP: 5’-terminal oligopyrimidine
P-bodies: Processing bodies
HD: Huntington’s Disease
AD: Alzheimer’s Disease
SG: Stress granule
AUC: Area under the curve
FeIMAC: Iron immobilized metal affinity chromatography
RT: Room temperature
SP: Sample prep
CAA: Chloroacetamide
DTT: Dithiothreitol
DDA: Data dependent acquisition
IACUC: Institutional Animal Care and Use Committee
