# Supplementary figures and images for: Broad proteomics analysis of seeding-induced aggregation of α-synuclein in M83 neurons reveals remodeling of proteostasis mechanisms that might contribute to Parkinson’s disease pathogenesis
Source: Mol Brain. 2024 May 22;17:26. doi: 10.1186/s13041-024-01099-1 (PMC11110445; doi:10.1186/s13041-024-01099-1)

A.)

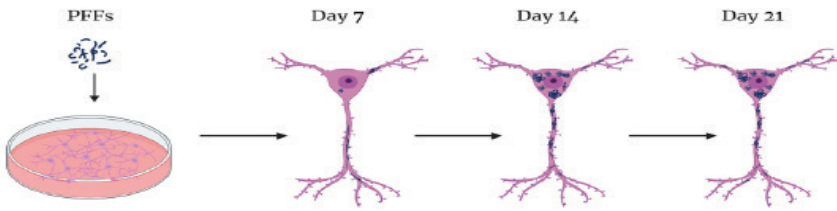

B.)

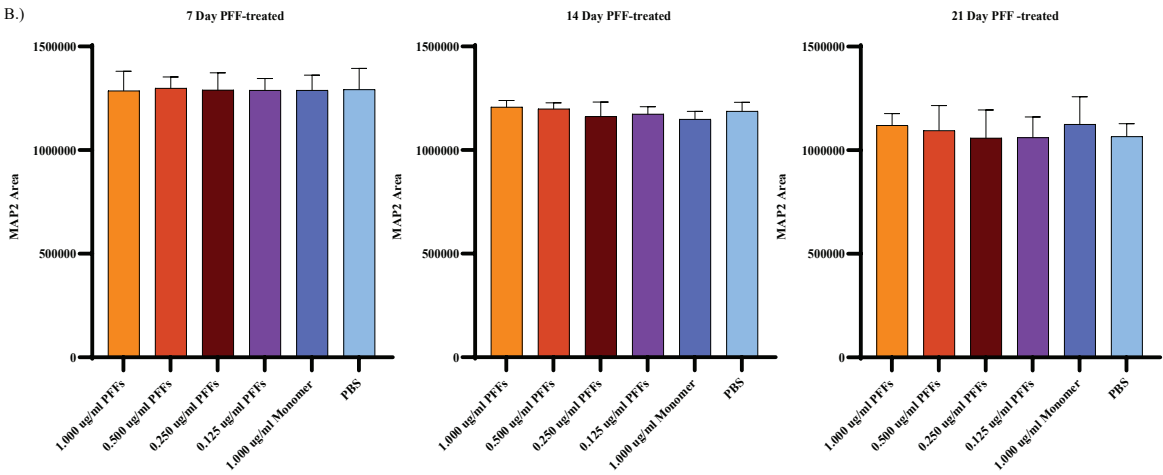

C.)

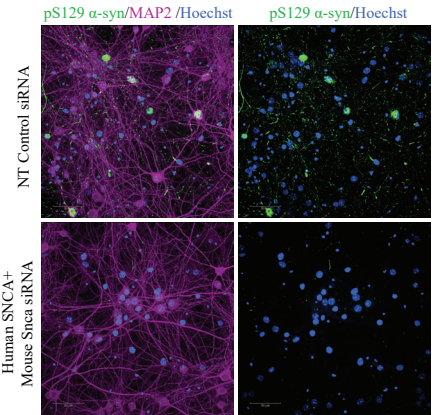

D.)

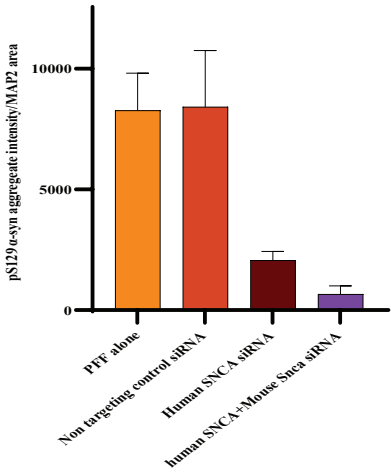

Figure S1.

A.)

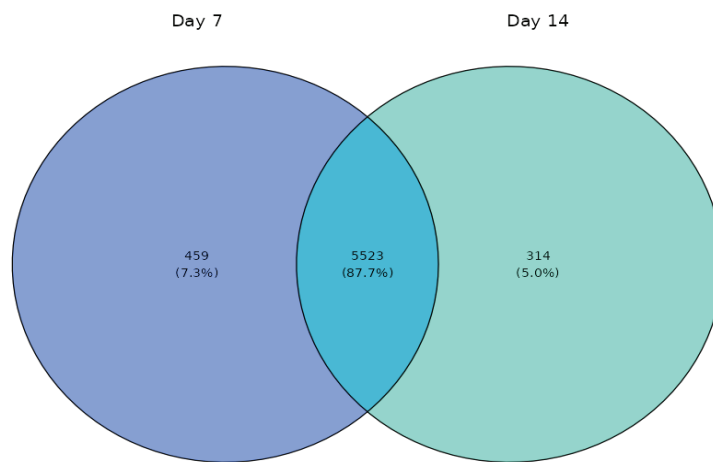

B.)

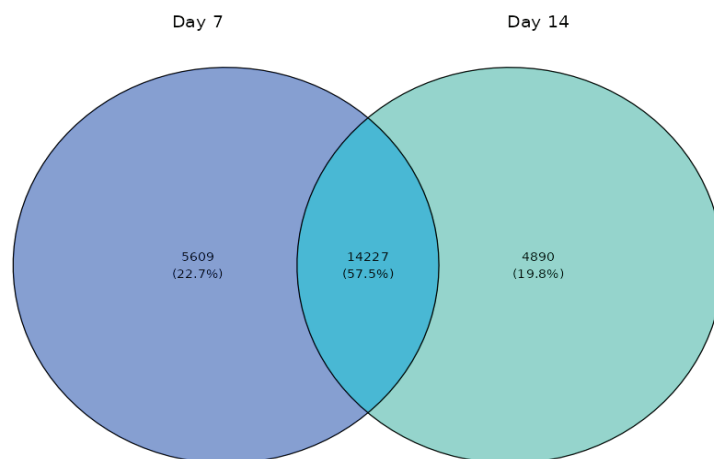

Figure S2.

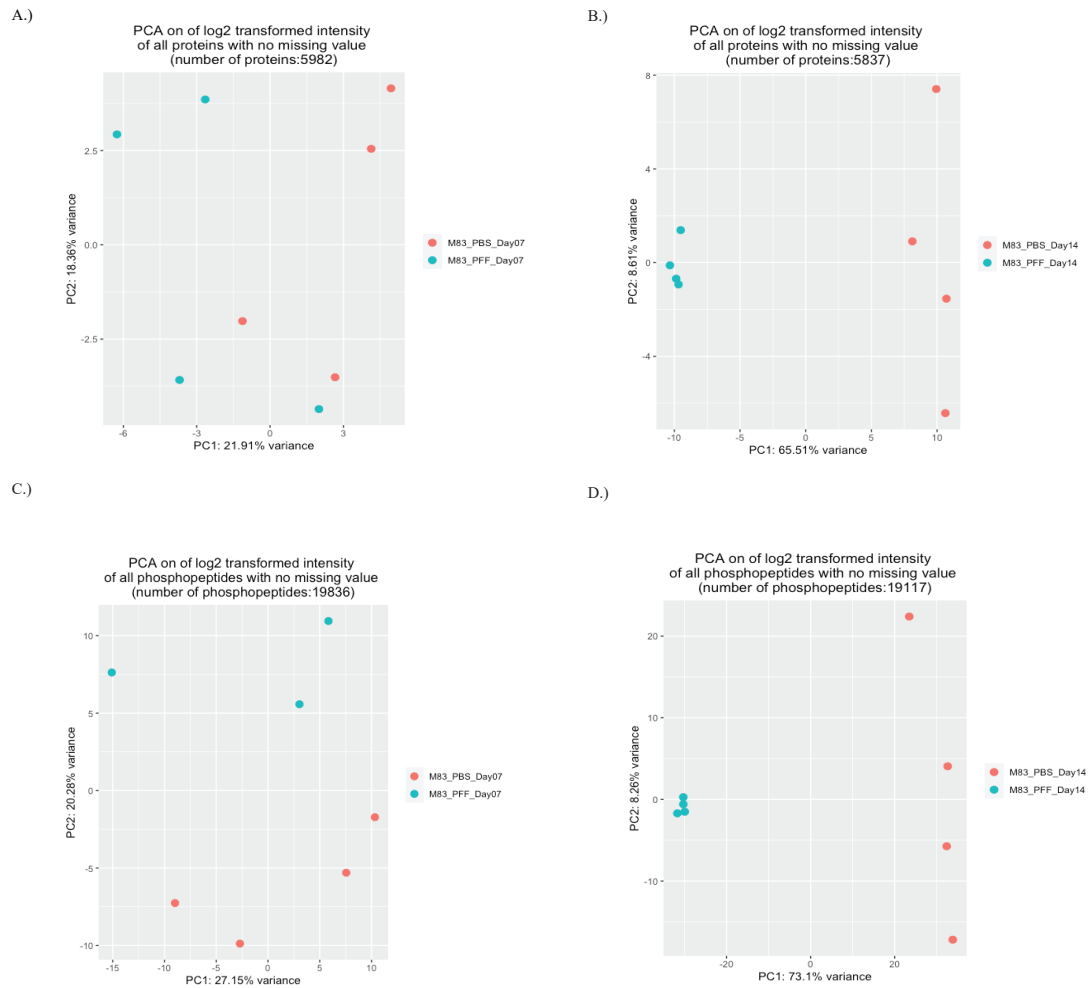

Figure S3.

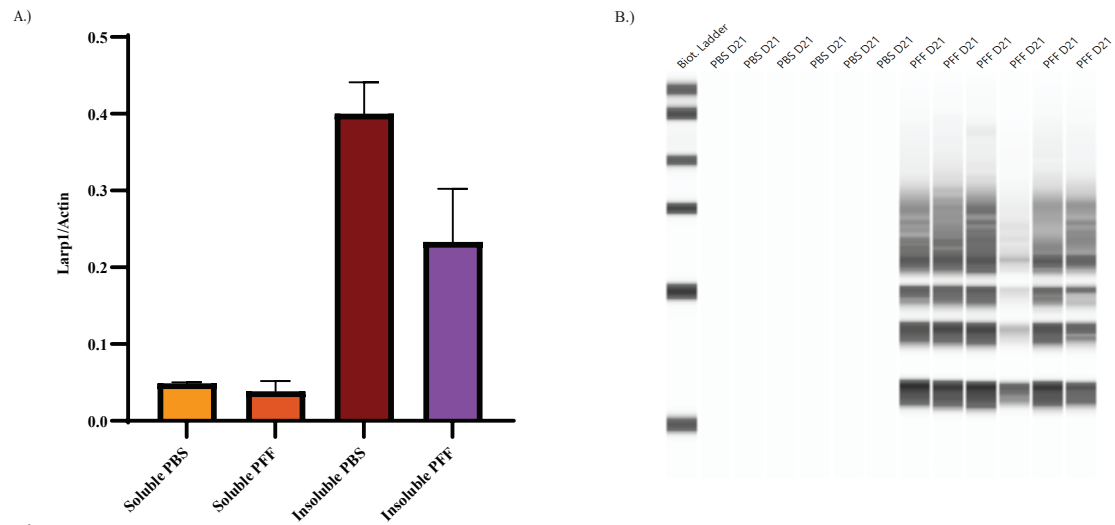

Figure S4.

Supplement: Supplementary file 1 — Supplementary Material 1: S1. Experimental paradigm for α-syn PFF treatment in M83 primary neurons. (A) Schematic of α-syn PFF treatment time course in M83 primary neuronal cultures. α-syn PFFs, monomer and PBS were added to neurons at DIV7 for 7, 14 and 21 days. (B) No significant changes in neuronal viability observed by quantification of MAP2 area at 7, 14 or 21 days with α-syn PFF treatment compared to α-syn monomer or PBS treatment. (C) shows representative images with non-targeting control siRNA and human SNCA and mouse SNCA siRNA treatment in M83 neurons stained with pS129 α-syn (green), MAP2 (purple) and nucleus with Hoechst (blue). Scale bars, 50 μm. and (D) shows quantification using high-content image analysis, which indicated that knockdown of human SNCA and mouse SNCA reduced PFF-induced pS129 α-syn aggregation. (N=5 replicates). S2. Number of proteins identified at day 7 and day 14 in total and phospho-proteomic analysis in total lysates of M83 neurons treated with α-syn PFFs. The number of proteins identified at day 7 and day 14 are summarized with Venn diagrams in A (total proteins) and B (phosphor-peptides) A high overlap at two timepoints was observed for both total proteins and phosphor-peptides. A total of 5523 proteins (87.7%) and 14,227 unique phosphopeptides (57.5%) were identified at both day 7 and day 14 timepoints. S3. Principal Component Analysis (PCA) of each individual time point in total and phospho-proteomic analysis in total lysates of M83 neurons treated with α-syn PFFs. Principal Component Analysis (PCA) was conducted at day 7 and day 14 with PFF and PBS treatments for both total (A, B) and phospho-proteomics (C, D). We observed clustering of replicates and separation by treatment. S4. (A) WES analysis quantification of soluble and insoluble fractions isolated from M83 mice. Data are mean±SD (B) M83 neurons treated with α-syn PFFs or PBS for 21± 1 days were sequentially extracted with 1% Triton X-100 followed by 2% SDS. Western blot [file 13041_2024_1099_MOESM1_ESM.pdf]
